# Compliance of amblyopic patients with occlusion therapy: A pilot study

**DOI:** 10.4103/0974-620X.53035

**Published:** 2009

**Authors:** Sana Al-Zuhaibi, Iman Al-Harthi, Pascale Cooymans, Aisha Al-Busaidi, Yahya Al-Farsi, Anuradha Ganesh

**Affiliations:** Department of Ophthalmology, College of Medicine and Health Sciences, Sultan Qaboos University, Muscat, Oman; 1 Department of Family Medicine and Public Health, College of Medicine and Health Sciences, Sultan Qaboos University, Muscat, Oman

**Keywords:** Amblyopia, compliance, occlusion therapy, self-reported efficacy

## Abstract

**Background:**

Increasing evidence shows that good compliance with occlusion therapy is paramount for successful amblyopia therapy.

**Purpose:**

To study the degree of compliance and explore factors affecting compliance in patients undergoing occlusion therapy for amblyopia in our practice.

**Design:**

Nonrandomized clinical intervention study.

**Materials and Methods:**

A total of 31 families with a child (aged 2-12 years), undergoing unilateral amblyopia treatment at the pediatric ophthalmology clinic of Sultan Qaboos University Hospital, Oman, were recruited for this one month study. Parents were interviewed and completed a closed-ended questionnaire. Clinical data including, visual acuity, refraction, diagnosis and treatment, for each patient was collected from the hospital chart and was entered in a data collection sheet. Compliance with occlusion therapy was assessed by self-report accounts of parents and was graded into good, partial, or poor. Association between various factors and degree of compliance was studied using logistic regression modeling.

**Results:**

Only 14 (45%) patients showed good compliance to occlusion therapy. 17 (55%) patients were noncompliant. Improvement in visual acuity strongly correlated with compliance to patching (*P* = 0.008). Other variables that were studied included, age at onset of therapy; gender; degree of amblyopia; type of amblyopia; use of glasses; and compliance with glasses. These did not emerge as significant predictors of compliance. All but one family with poor compliance stated that the main challenge in following the recommendation to patch for requisite hours was in getting their child to cooperate. Only in one instance, the family cited nonavailability of patches as the main hindrance to compliance. 10/31 (32%) families expressed a desire for more information and 18/31 (58%) parents did not understand that amblyopia meant decreased vision.

**Conclusion:**

Poor compliance is a barrier to successful amblyopia therapy in our practice. Improvement in visual acuity is associated with better compliance with patching. Parents find it difficult to comprehend and retain verbal explanations of various components regarding occlusion therapy for amblyopia. Future study with a larger sample of patients is recommended to investigate the factors affecting compliance with amblyopia therapy and determine predictors for poor compliance.

## Introduction

In 2002, the prevalence of childhood blindness in Oman was reported to be 0.08%.[1] It is estimated that the cumulative number of blind-person-years worldwide due to childhood blindness ranks second only to the cumulative number of blind-person-years due to cataract blindness,[2] indicating a need to attempt effective control of childhood visual problems. Vision 2020, a global initiative launched jointly by the World Health Organization and the International Agency for the Prevention of Blindness, has one of its aims as elimination of avoidable causes of blindness in children. Amblyopia is one of the most common causes of visual impairment in children with a prevalence varying between 0.2 and 5.3%, depending on the subsets of the population studied.[3,4] In Oman, amblyopia prevalence has been reported to be 0.3%.−0.92%[5,6] It has been proved that there is an increased risk of vision loss in the better eye in patients with amblyopia, which may add to the visual impairment caused by amblyopia and decreasing quality of life in adulthood.[7]

Amblyopia is derived from the Greek word, 'amblyos' meaning dull and 'opia' meaning vision, refers to a decrease in best-corrected visual acuity in an eye having no organic pathology. In the multicenter Amblyopia Treatment Study (ATS), out of 409 patients, 37% were anisometropic, 38% were strabismic, and combined mechanism was the etiology in 24% patients.[8] The ATS did not recruit patients with deprivation amblyopia.

Amblyopia must be detected early and the pathology must be addressed prior to the treatment for amblyopia. Deprivation amblyopia produced by media opacities such as cataract is typically the most severe form. It has also been shown that amblyopia does not resolve without treatment.[9] During the last decade, the Pediatric Eye Disease Investigative Group (PEDIG) has initiated and completed several multicentered controlled clinical trials evaluating different treatments for amblyopia.[10-14] The impact of this fundamental research proving beyond doubt that amblyopia treatment is effective, has been dramatic with the establishment of vision screening programs, both preschool and again on school entry. Thus, by 5 or 6 years of age, the vast majority of school entrants in countries such as UK and US, have been screened for amblyopia two, three, or more times.[9]

Compliance is referred to as correspondence between recommendations from the healthcare provider and the patients' actual dosage.[15] Noncompliance to amblyopia therapy through self-reports has limitations. It has various connotations and may be related to duration of occlusion and the method of occlusion. The patient or care-giver may be unable to describe the occlusion regimen. Poor compliance has been stated to be an important obstacle for providing effective healthcare in long-term treatments. In amblyopia also, "compliance" with treatment has been established as an important factor affecting the final visual outcome,[16] and poor compliance has been shown to be a risk factor for treatment failure.[17] A study employing electronic monitoring to investigate predictors of noncompliance implicated a low level of parental education, and poor acuity at the start of treatment as predictors of low compliance.[18] However, another study evaluating psychosocial and clinical variables influencing parental compliance with occlusion therapy, concluded that severity of visual impairment did not affect compliance; perceived self-efficacy was positively-associated and perceived prohibition of the child's activities was negatively-associated with compliance.[19] A third study stated that difficulties encountered by parents was the most important factor influencing compliance to amblyopia therapy and emphasized that, measures should be undertaken to improve compliance and address these issues. Low compliance has also reported to be associated with social deprivation,[20] and younger age at presentation.[4]

We conducted a nonrandomized clinical intervention study to obtain preliminary data about the level of compliance and factors influencing compliance in patients undergoing therapy for amblyopia in our practice.

## Materials and Methods

Families with a child (aged 2–12 years), who were undergoing unilateral amblyopia treatment for at least three months and attended the pediatric ophthalmology clinic during September−October, 2008, were recruited to participate in the study. Parents were explained about the study and informed consent for participation was obtained. This pilot study was approved by the Departmental Research Committee.

All children received a routine orthoptic and ophthalmic examination, and explanation of their eye condition and treatment by the healthcare providers. Visual acuity was measured in our clinic using the O-test or Kolt-test.[21] Amblyopia was considered when a difference in the best-corrected visual acuity between the two eyes of two or more lines was observed. The number of patching hours was decided according to the age of patient and degree of amblyopia [Table 1]. All patients were patched with a commercially available opaque patch [Nexcare Opticlude Orthoptic Eye Patch; 3 M Nexcare, 3 M Corporate Headquarters 3 M Center, St. Paul, MN 55144-1000, USA], over the nonamblyopic eye. Children with developmental disorders or those with other ocular problems contributing to visual impairment were excluded from the study.

**Table 1:** Protocol for occlusion therapy of amblyopia

*Degree of Amblyopia*	*Age (0–7 years)*	*Age (8–12 years)*
Mild and moderate amblyopia (VA = 0.25–0.9)	2 hours/day patching*	4–6 hours/day patching*
Severe amblyopia (VA = < 0.25)	4–6 hours/day patching*	Constant patching*

* All patching was accompanied by one hour of near visual activities such as: play station, computer, homework, art

**Figure 1:**
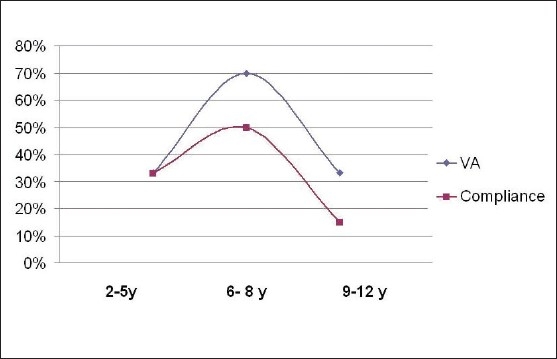
Correlation of visual acuity progress and age with compliance to occlusion therapy. Improvement in visual acuity correlated with good compliance (*P* = 0.001)

Parents (one or both parents) of children were interviewed in the clinic by an Arabic speaking investigator (IAH) and a closed-ended questionnaire was completed [Figure 1]. The questions explored parents' perception regarding amblyopia and its treatment; their experiences regarding patching; and self-perceived view regarding compliance. The questionnaire was developed following a review of literature, pilot interviews, and discussions within the study team.

Compliance was graded as good or partial, based on whether the number of hours patched was equal to or within 50% of the recommended hours. If no patching was done or the number of patching hours was below 50% of the recommended hours, then it was recorded as poor.

Clinical data extracted from the patient's chart included demographic details, age of the child, visual acuity at onset, visual acuity after onset of patching therapy, refraction, compliance with glasses, type of amblyopia, initial diagnosis, and treatment. Strabismic amblyopia was defined as amblyopia in the presence of a heterotropia at distance or near fixation in the absence of any anisometropia meeting the criteria for a combined mechanism amblyopia. Anisometropic amblyopia was defined as amblyopia in the presence of anisometropia that was 1D or greater in spherical equivalent, or a 1.5 D or greater difference in astigmatism between both the eyes that persisted for at least four weeks after spectacle correction; in the absence of any measurable heterotropia at distance or near. Deprivation amblyopia was caused by a media opacity such as corneal opacity or cataract that persisted after correction of the underlying problem and refractive error. Combined mechanism amblyopia included patients with a mix of either or all of the above mechanisms of amblyopia.

Amblyopia was classified as mild/moderate/severe, based on whether visual acuity in the amblyopic eye at presentation was 0.5−0.9, 0.2−0.5, or < 0.2, respectively. All information was entered in a data collection sheet.

### Statistics

Statistical analyses were performed using SPSS 16.0 for Windows software (SPSS Chicago, Illinois, USA). We produced descriptive statistics and frequency distribution plots for all parameters included in the analyses. The associations between compliance to occlusion therapy and selected predictors, were evaluated by conducting logistic regression models. We first evaluated the degree and significance of association by cross-tabulation using 2 x 2 contingency tables. We produced odds ratios (OR), 95% confidence interval (CI), and *P* values, by applying Chi-squared (χ^2^) tests and Cochrane's and Mantel-Haenszel statistics. Afterwards, we entered significant predictors in binary logistic regression models in a forward stepwise method. We evaluated the significance of associations according to OR, 95% CI, and *P* values that were produced by the regression models. Two-tailed *P* values considered throughout and the statistical significance were taken at *P* < 0.05.

## Results

A total of 31 families with children participated in our study; 16 (52%) were boys and 15 (48%) were girls. The median age at presentation was six years (range, 2−12 years). All patients had undergone occlusion therapy for a minimum period of three months.

The study group consisted 15 strabismic (48%), 4 anisometropic (12%), 5 deprivation (16%), and 7 combined mechanism (24%) amblyopic patients. The level of amblyopia was mild in 14 (45%), moderate in 8 (26%), and severe in 9 (29%) patients [Table 2].

**Figure 2:**
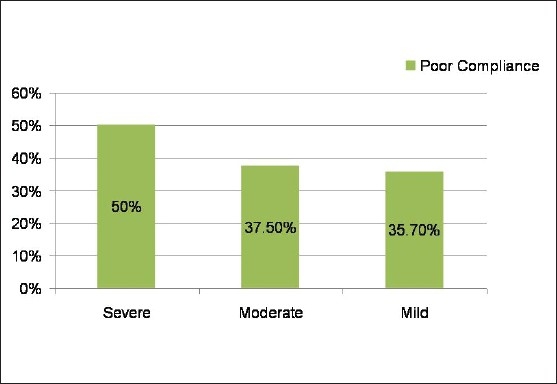
Correlation of severity of amblyopia with compliance to occlusion therapy. There is a trend towards higher incidence of poor compliance in patients with severe amblyopia (*P* = 0.97; not significant)

Compliance level was good in 14 (45%), partial in 4 (13%), and poor in 13 (42%) patients. Improvement in visual acuity was strongly correlated with compliance to patching (*P* = 0.008) [Figure 1]. Other studied variables including, age at onset of therapy; gender; degree of amblyopia (or level of visual acuity at onset); type of amblyopia; use of glasses (*P* = 0.307); and compliance with glasses (*P* = 0.151), did not emerge as significant predictors of compliance. However, there was a trend towards higher incidence of poor compliance in patients with severe amblyopia [Figure 2] and anisometropic amblyopia.

In most patients, the mother was responsible for the treatment at home. In eight patients (26%) the father also participated in the care of the child. In one instance, in the case of an uncooperative child, the school teacher's help was recruited to enforce therapy.

All but one family with poor compliance stated that the main challenge in following the recommendation to patch for requisite hours was poor cooperation on the part of the child. Only in one instance, the family cited nonavailability of patches as the main hindrance to compliance.

Twenty-three of 31 children (74%) were prescribed glasses. When evaluated as a predictor for poor compliance presence of glasses did not significantly correlate with poor compliance. Compliance with glasses was very good in our cohort and only seven of 23 patients (30%) showed poor compliance. Of these seven, five were girls.

Parents of ten children expressed that they wanted more information about their child's eye condition. Eighteen (58%) parents did not understand that amblyopia meant decreased vision. However, 29 (93%) parents felt that occlusion therapy would benefit their child.

Almost all parents had to employ certain strategies to ensure their child's cooperation. The most common ones were rewarding and explaining the need for patching to the child.

Five children with poor compliance (38%) were admitted into the hospital for short periods to ensure compliance. The clinical profile of these children is presented in Table 2. All five children had moderate-severe amblyopia. Four of the five children (80%) showed improvement in visual acuity and improved compliance to occlusion therapy after the admission.

**Table 2:** Variables studied to determine effect on compliance with occlusion therapy. [(N = 31) total patients undergoing therapy]

*Variables*	*Details*
Gender	
Male	16 (52%)
Female	15 (48%)
Age at onset of occlusion Type of amblyopia	3.9 years (3 months-10 years)
Anisometropia	15 (48%)
Strabismic	4 (12%)
Deprivation	5 (16%)
Combined	7 (24%)
Degree of amblyopia	
Mild	14 (45%)
Moderate	8 (26%)
Severe	9 (29%)
*Improvement in vision	
Present	10 (32%)
Absent	21 (68%)
Use of Glasses	
Yes	23 (75%)
No	8 (25%)
Compliance with glasses	
Yes	16 (70%)
No	7 (30%)
Admission for compliance	n = 5 children
Age range	4–12 years
Type of amblyopia	
Strabismic	2
Deprivation	3
Depth of amblyopia	
Moderate	2
Severe	3
Outcome	
Improved compliance and vision	4

* Improvement in visual acuity was strongly correlated with compliance to patching (*P* = 0.008).

## Discussion

Amblyopia is the most common cause of monocular visual impairment among children, young, and middle-aged adults.[4] Patching of good eye is the mainstay of therapy. Many studies have shown that compliance to patching or occlusion therapy is a major factor affecting the outcome of treatment. The degree of compliance and factors affecting compliance in patients being treated for amblyopia in our practice are currently unknown.	

Our study comprised 31 families with children (aged between 3 months−10 years), who were undergoing occlusion therapy for all types of unilateral amblyopia for at least three months. There were more patients with strabismic amblyopia (48%) compared to anisometropic (12%) or deprivation (16%) amblyopia in our study sample. Mild amblyopia was more prevalent (45%) compared to moderate (26%) or severe (33%) ones.

We observed that only 45% parents in our practice showed good compliance with patching recommendations. Compliance with occlusion therapy was calculated on the basis of self-reported efficacy. A study similar to ours reported a compliance level of 54%.[19] Compliance with occlusion therapy for amblyopia can also be measured electronically by means of an occlusion dose monitor (ODM)[22] Studies using ODMs have also revealed low compliance rates ranging from 48−58%[23,24] It appears that noncompliance with occlusion therapy is widely prevalent, substantial, and a major factor leading to treatment failure.

In our study, the only improvement in visual acuity among all the variables assessed had a significant influence on compliance. Age at onset of therapy, gender, degree of amblyopia (or level of visual acuity at onset of therapy), type of amblyopia, use of glasses, and compliance with glasses, did not emerge as significant predictors of compliance. It is possible that a larger number of patients would allow better subgroup analysis and may have given different results. The trend of anisometropic amblyopia being correlated with poor compliance is difficult to explain. We initially felt that this could be due to the delayed age at presentation that is usually seen in patients with anisometropic compared to other types of amblyopia;[25] possibly secondary to a bias due to other types of amblyopia being usually associated with a cosmetically noticeable defect which drives care, whereas anisometropic patients have no identifiable defect.[26] However, looking at the patients' ages, it was observed that patients in the three different types of amblyopia had a near similar average age at presentation (5 years for anisometropia, 3.8 years for strabismus, and 4 years for deprivation amblyopia). Further, patients in the anisometropic group had less severe amblyopia than the other two groups. Therefore, we are unable to account for the higher prevalence of poor compliance in the anisometropic group and await the results from a larger ongoing study to validate this finding.

Research in other areas of medicine has shown that the understanding level can have a direct effect on the level of compliance. We observed that one-third parents in our study were dissatisfied with information given to them; although, more than 90% were highly sensitive to the credibility of the treatment, approximately 50% were confused by the information given in the clinic and had poor understanding about the disease. We believe that written information about the critical period, importance of occlusion, and potential negative consequences of not treating amblyopia, will be more effective in improving parental understanding and subsequent compliance.[27] Knowledge of the critical period and the reducing prognosis with age would help induce a sense of urgency in the parents and prevent the treatment being delayed by them, until a time, when they consider the occlusion would be more easily tolerated. In our study, parents described a range of strategies for facilitating patching, including explanation, rewards, and enlisting the help of others.

Our study has certain limitations. Firstly, we cannot be certain of the extent to which parental self-report of compliance is accurate. Indeed, parents may overestimate levels of patching (even though assured of anonymity), and reports are also subject to accurate recall. Secondly, the different types of amblyopia in our study sample occurred in varying proportions. Also, the three groups varied considerably with respect to age at presentation, and severity of amblyopia or baseline acuity. Since compliance might be altered by all these variables, it would be ideal to have matching proportions and some similarity between groups. Finally, as all pilot studies, our numbers are too small to obtain statistical significance.

Despite these limitations, this pilot study has shown that compliance to occlusion is a major problem in our practice that can impact outcome of amblyopia therapy. Improved visual acuity was seen to significantly influence the compliance; if parents were made aware of improvements in visual acuity, compliance might be enhanced. Parental education, particularly with the use of written material providing explanation regarding the critical period, importance of occlusion, and the potential negative consequences of not treating amblyopia should be implemented. We recommend a larger study with a bigger sample size avoiding the aforementioned limiting factors to verify the results of the current study, and investigate the influence of various clinical and psychosocial variables on compliance with occlusion therapy in amblyopia.
